# Genetic Profiles of Purine, Uric Acid, Superoxide Dismutase, and Growth in Thai Slow-Growing Chickens

**DOI:** 10.3390/ani14243658

**Published:** 2024-12-18

**Authors:** Wuttigrai Boonkum, Vibuntita Chankitisakul, Srinuan Kananit, Veeraya Tuntiyasawasdikul, Vatsana Sirisan, Wootichai Kenchaiwong

**Affiliations:** 1 Department of Animal Science, Faculty of Agriculture, Khon Kean University, Khon Kean 40002, Thailand; wuttbo@kku.ac.th (W.B.); vibuch@kku.ac.th (V.C.); srinka@kku.ac.th (S.K.); veerayat@kkumail.com (V.T.); 2 Network Center for Animal Breeding and Omics Research, Khon Kaen University, Khon Kaen 40002, Thailand; 3 Small Ruminant Research Unit, Faculty of Veterinary Science, Mahasarakham University, Mahasarakham 44000, Thailand; siri.vatsana@gmail.com

**Keywords:** growth characteristics, purine content, superoxide dismutase, genetic parameter, Thai native chicken

## Abstract

The objective of this study was to estimate genetic parameters and genetic correlations between growth characteristics and purine and uric acid in the breast and liver and superoxide dismutase (SOD) in the blood. The growth characteristics included body weight (BW) at hatching (BW0), BW at 2, 4, 6, 8, and 10 weeks of age, average daily gain (ADG) at 0–2, 2–4, 4–6, 6–8, and 8–10 weeks of age, and breast circumference at 6, 8, and 10 weeks of age (BrC6, BrC8, and BrC10) were recorded from 300 Thai native chickens (Shee breed). In total, 30 chickens (15 males and 15 females) were randomly euthanized to collect breast meat, liver, and blood samples to determine the purine content. A multiple-trait animal model and an average information-restricted maximum likelihood (AI-REML) were used to estimate the variance components and genetic parameters. The estimated heritability values for all growth traits were moderate and ranged from 0.304 to 0.485, 0.270 to 0.335, and 0.286 to 0.314 for BW, ADG, and BrC, respectively. The estimated heritability values for various biochemical traits, including purine content, uric acid, and SOD levels, were low to moderate and ranged from 0.035 to 0.143, and 0.050 to 0.213 in breast meat and liver, respectively. In genetic correlations, total purine content showed a strong negative correlation with growth traits, whereas uric acid and SOD levels exhibited varying degrees of correlation with BW and ADG. These results highlight the importance of genetic parameters between growth and biochemical traits in Thai native chickens and provide valuable insights for breeding programs aimed at improving growth performance and meat quality. This study indicated the potential use of heritability values and genetic correlations to enhance selective breeding strategies using the multiple-trait genetic evaluation method for optimal trait combinations in poultry.

## Simple Summary

Improving growth potential and ensuring good meat quality in native chickens were among the key goals in industrial poultry production. This study estimates genetic parameters and correlations of growth, purine content, uric acid, and superoxide dismutase (SOD) activity in Thai native chickens. Findings reveal moderate heritability for growth traits, while biochemical traits showed lower heritability, suggesting potential for selective breeding. The study provides insights to enhance meat quality and antioxidant capacity while balancing growth performance, aiding in breeding programs aimed at sustainable development of Thai native chickens.

## 1. Introduction

Developing faster growth characteristics in poultry is a significant goal in poultry production systems because it allows farmers to raise chickens for multiple production cycles, thereby increasing their income [1,2]. However, enhancing growth potential can negatively impact the quality of chicken meat, particularly the levels of bioactive compounds such as purines and uric acid in meat and organs. These compounds are undesirable for consumers because they can cause gout [3]. Fast-growing chickens have a high metabolic rate [4] and are selectively bred to rapidly develop muscle tissue, which requires increased protein synthesis [5]. Because purines are a natural component of proteins, the accelerated growth rate in these chickens may lead to higher purine content in their muscle tissue than in slow-growing chickens [5,6,7]. Additionally, high growth and metabolic rates in chickens lead to increased stress levels and higher accumulation of reactive oxygen species (ROS) [8]. Research has shown that the levels of antioxidant enzymes, such as superoxide dismutase (SOD) and malondialdehyde (MDA), as well as the antioxidant capacity (AOC) and glutathione peroxidase (GPx), vary according to the breed and growth rate of chickens [9,10,11]. These enzymatic reactions are crucial for the defense against oxidative stress, as superoxide radicals are highly reactive and can damage cellular components, including proteins, lipids, and DNA [12]. Therefore, maintaining adequate levels of these antioxidant enzymes is essential to manage stress and minimize oxidative damage in chickens.

Thai native chickens are highly valued for their distinctive meat quality, including unique texture and flavor, appealing to niche consumer groups. Despite their slower growth rate, their ability to retain desirable meat characteristics and bioactive compounds presents opportunities for genetic improvement, supporting sustainable poultry production [13,14,15]. They exhibit superior antioxidant properties and lower purine levels compared to commercial broilers, highlighting their potential as a functional food [6]. However, their primary limitation is a slow growth rate, which hinders their competitiveness as commercial broilers. Consequently, previous research on slow-growing chickens has primarily focused on enhancing their growth potential [16,17,18], often neglecting the quality of meat in terms of essential bioactive compounds such as purine content and stress-related factors. This may inadvertently result in the accumulation of high levels of purine compounds in meat, similar to commercial broiler production. Although methods such as rinsing and cooking can reduce purine compounds in chicken meat [19,20], these approaches do not address the root cause of this problem. Genetic selection may be an effective and sustainable solution to this problem. The aim of this study was to estimate the genetic parameters for growth, purine content, uric acid, and SOD in Thai native chickens. Upon obtaining the results of this study, we aim to contribute to the sustainable development of indigenous chicken genetics by promoting optimal growth rates and minimizing purine accumulation. Our ultimate goal is to develop indigenous chicken products that can be regarded as healthy and nutritious food options for consumers in the future.

## 2. Materials and Methods

The research conducted at the Khon Kaen University was reviewed and approved by the Institutional Animal Care and Use Committee of the Ethics of Animal Experimentation Guidelines established by the National Research Council of Thailand (No. IACUC-KKU-14/65; 20 January 2022). This study was conducted at the experimental farm of the Network Center for Animal Breeding and Omics Research, Faculty of Agriculture, Khon Kaen University, Thailand.

### 2.1. Animal Management and Data Collection

In total, 300 Thai native chickens (Shee breed) were genetically selected as part of previous breeding improvement programs. In addition, the pedigree data of the chickens in this study were recorded to ensure accurate genetic evaluations. All chickens were bred and managed according to Thai native chicken farming standards and raised in an open house system. Each chicken was fed ad libitum with a commercial diet divided into two phases: the initial phase (21% crude protein, 5% crude fiber, 3100 kcal metabolizable energy) for the first 4 weeks after hatching (0–4 weeks of age), and the growth phase (19% crude protein, 5% crude fiber, 3200 kcal metabolizable energy) from the 4th week to the end of the experiment (10 weeks of age). After hatching, all chicks were numbered and marked on their legs for identification until they were 4 weeks old; thereafter, they were marked on their wings. For the first 2 weeks, the chickens were raised using a 100-watt heating lamp. The light program was divided into two phases: from hatching to week 4, they received 24 h of light and 0 h of darkness; and from weeks 5 to 10, they were exposed to natural light. The daylight duration ranged from 11 to 13 h, varying with the season. Ambient temperatures were recorded between 28 °C and 34 °C. Birds were provided with ad libitum access to water via nipple drinkers, while feed was supplied using circular feeders.

Individual growth characteristics were recorded during the data collection process, including body weight (BW) at hatching (BW0) and at 2, 4, 6, 8, and 10 weeks of age (BW2, BW4, BW6, BW8, and BW10, respectively). The average daily gain (ADG) was calculated for the intervals 0–2, 2–4, 4–6, 6–8, and 8–10 weeks (ADG0–2, ADG2–4, ADG4–6, ADG6–8, and ADG8–10, respectively). Additionally, breast circumference (BrC) was measured at 6, 8, and 10 weeks of age (BrC6, BrC8, and BrC10, respectively). For purine content and SOD analysis, thirty chickens (15 males and 15 females) were randomly euthanized each week from week 6 to week 10, the final week of the experiment, to collect breast, liver, and blood samples. Breast meat samples were randomly collected from six locations on both sides of the breast (top, middle, and bottom of the right and left sides) using a lancet and stored in plastic bags. Liver tissues were collected and stored in a similar manner. The samples were then prepared for further analysis. The content of purines—adenine, guanine, xanthine, and hypoxanthine—and uric acid (calculated based on purine content) in the breast and liver, as well as SOD in the blood, were examined. Breast meat and liver samples were preserved by freezing in liquid nitrogen and stored at −20 °C to determine purine and uric acid content. Approximately 1 mL of blood was collected from the brachial vein, and the serum was stored at −20 °C for future SOD analysis.

### 2.2. Purine Content and SOD Analysis

The purine (adenine, guanine, hypoxanthine, xanthine) and uric acid in breast meat and liver were analyzed using HPLC (Shimadzu LC20A, Shimadzu Corporation, Tokyo, Japan). Samples (~500 mg) were homogenized with deionized water and perchloric acid, incubated at 95 °C for 1 h, neutralized with potassium hydroxide, and centrifuged. The supernatant was filtered and injected into the HPLC system equipped with an Asahipak GS-HQ 320HQ column. A sodium phosphate buffer (pH 2.5) was used as the mobile phase, with a flow rate of 0.6 mL/min over 35 min. Results were averaged from two replicates, and purine content was calculated as the total of all derivatives [21,22]. Meanwhile, SOD activity was measured following the method of Ratchamak et al. [23]. Plasma was mixed with a cytochrome c solution, xanthine, and xanthine oxidase. Absorbance at 550 nm was monitored every 5 min to measure cytochrome c reduction caused by superoxide anions. The activity was calculated as the concentration needed to achieve a 50% reduction in SOD activity, comparing sample rates to a blank.

### 2.3. Genetic Analysis

Before conducting the genetic analysis, the raw data were verified for normality using the Shapiro–Wilk test and for homogeneity of variance using Levene’s test with the Proc UNIVARIATE procedure in SAS software version 9.0. Outliers were eliminated prior to the analysis. Data on growth traits, purine content, uric acid, and SOD were subjected to a multifactor analysis of variance (ANOVA), considering sex, chicken hatch set, and breed group (blood levels of Shee breed were measured at 100%, 50%, and 25%). This analysis used the general linear model (GLM) procedure for unbalanced data in SAS software version 9.0. Multiple pairwise comparisons were conducted using Scheffe’s test (*p* < 0.05) when significant differences were detected. All data are expressed as mean values ± standard deviations. Variance components and genetic parameters such as heritability ($h^{2}=\frac{\sigma_{a}^{2}}{\sigma_{a}^{2}+\sigma_{e}^{2}}$, where $\sigma_{a}^{2}$ and $\sigma_{e}^{2}$ = additive genetic and residual variances), genetic correlation ($r_{g}=\frac{{\mathrm{COV}\sigma }_{\mathrm{aij}}}{\sqrt{\sigma_{\mathrm{ai}}^{2}{*\sigma }_{\mathrm{aj}}^{2}}}$, where ${\mathrm{COV}\sigma }_{\mathrm{aij}}$ = covariance between trait i and j, $\sigma_{\mathrm{ai}}^{2}{\;\mathrm{and}\;\sigma }_{\mathrm{aj}}^{2}$ = additive genetic variances of traits i and j), and phenotypic correlation ($r_{p}=\frac{{\mathrm{COV}\sigma }_{\mathrm{pij}}}{\sqrt{\sigma_{\mathrm{pi}}^{2}{*\sigma }_{\mathrm{pj}}^{2}}}$, where ${\mathrm{COV}\sigma }_{\mathrm{pij}}$ = phenotypic covariance between trait i and j, $\sigma_{\mathrm{pi}}^{2}{\;\mathrm{and}\;\sigma }_{\mathrm{pj}}^{2}$ = phenotypic variances of traits i and j) were estimated using a multiple-trait animal model and the average information-restricted maximum likelihood (AI-REML) approach with the BLUPF90 family program [24]. The models used for the analysis were as follows:

Y=Xb+Za+ε

where Y is the vector corresponding to the observation values; X and Z are incidence matrices related to fixed and random effects, respectively; b is the vector of fixed effects, including the chicken hatch set and sex; a is the vector of random additive genetic effects, assumed to be $a\sim N\left(0,A\sigma_{a}^{2}\right),$ where A is an additive relationship matrix and $\sigma_{a}^{2}$ is the additive genetic variance; and e is the vector of random residual effects assumed to be $e\sim N\left(0,I\sigma_{e}^{2}\right),$ where I is the identity matrix and $\sigma_{e}^{2}$ is the residual variance.

## 3. Results

### 3.1. Descriptive Statistics of Traits

The BW, ADG, BrC, purine content, uric acid in breast meat and liver, and SOD levels in the blood of Thai native chickens of different ages are summarized in Table 1 and Table 2. Growth traits recorded in this study included BW from hatching to 10 weeks of age. The BWs of Thai native chickens ranged from 35.47 g at hatching to 1834.56 g at 10 weeks of age, showing a significant increase over time. ADG was recorded every 2 weeks (ADG0–2, ADG2–4, ADG4–8, and ADG8–10), with values ranging from 21.63 g to 27.97 g. ADG2–4 was the lowest, similar to ADG0–2, whereas ADG4–8 and ADG8–10 did not show any significant differences (*p* < 0.05). BrC, measured at 6, 8, and 10 weeks increased significantly with age (*p* < 0.05). This study also examined purine and uric acid levels in native Thai chickens at 6, 8, and 10 weeks old. The liver consistently showed higher values than the breasts at all ages (*p* < 0.05). Purine and uric acid levels in the breast were higher at 6 weeks than at 10 weeks, except for guanine and xanthine, which showed no significant differences across the age groups (*p* < 0.05). The purine content in the liver was higher at 6 weeks than at 10 weeks, with significant differences in uric acid levels across all age groups. The highest uric acid levels were recorded at 6 weeks, with values decreasing thereafter (*p* < 0.05). Furthermore, SOD activity was the highest in chickens aged 6 weeks and decreased with age (*p* < 0.05).

### 3.2. Heritability Estimates

The estimated heritability of BW, ADG, and BrC is presented in Table 3. This study demonstrated that heritability estimates for BW, ADG, and BrC decreased with age. The estimated heritability values for all growth traits were moderate and ranged from 0.304 to 0.485, 0.270 to 0.335, and 0.286 to 0.314 for BW, ADG, and BrC, respectively. Heritability estimates for various biochemical traits in Thai native chickens were analyzed for breast meat and liver tissues, and SOD in the blood are presented in Table 4. The total purine content in breast meat at six weeks was 0.104, whereas it was 0.190 in the liver. At 8 and 10 weeks, the heritability of total purines in breast meat remained stable at 0.103, whereas it was 0.189 in the liver. The heritability of breast meat at 6 weeks was 0.086 for adenine content, compared to 0.115 in the liver. At 8 and 10 weeks, the heritability estimates for adenine in breast meat were 0.082 and 0.081, respectively, and those in the liver were 0.112 and 0.109, respectively. The heritability of guanine content in breast meat was 0.143 at 6 weeks, which decreased slightly to 0.138 and 0.134 at 8 and 10 weeks, respectively. In the liver, the heritability of guanine was higher (0.180 at 6 weeks, 0.173 at 8 weeks, and 0.168 at 10 weeks). For xanthine content, the heritability in breast meat was 0.036 at 6 and 8 weeks, and slightly lower at 0.035 at 10 weeks. In the liver, the heritability of xanthine was 0.071 at 6 weeks, 0.062 at 8 weeks, and 0.056 at 10 weeks. The hypoxanthine content in breast meat exhibited heritability estimates of 0.102, 0.100, and 0.098 at 6, 8, and 10 weeks, respectively. In contrast, the liver had lower heritability values of 0.069, 0.057, and 0.050 at 6, 8, and 10 weeks, respectively. When calculated as uric acid, the heritability in breast meat was 0.120 at 6 weeks and remained nearly constant at 0.119 at both 8 and 10 weeks. In the liver, the heritability of uric acid was significantly higher at 0.213, 0.200, and 0.184 at 6, 8, and 10 weeks, respectively. Regarding SOD in the blood, the heritability estimates were 0.290, 0.278, and 0.268 at 6, 8, and 10 weeks of gestation, respectively. From the results mentioned above, the heritability estimates for these traits were generally higher in the liver than in breast meat across all age groups, indicating a more substantial genetic influence on these biochemical traits in liver tissue than in breast meat. SOD levels in blood exhibited relatively high heritability, suggesting a substantial genetic component.

### 3.3. Genetic and Phenotypic Correlation Estimates

Genetic and phenotypic correlations between growth traits, purine content, and uric acid in breast meat and liver, and SOD in the blood are presented in Table 5 and Table 6. Growth traits, such as BWs at different weeks (BW0, BW2, BW4, BW6, BW8, and BW10), showed high positive genetic correlations with the ADG in the corresponding growth periods, ranging from 0.68 to 0.97. These results indicated that growth traits were genetically interrelated. Furthermore, the genetic correlations between BrC traits (BrC6, BrC8, and BrC10) and BWs were generally high, suggesting a strong genetic relationship between meat quality traits and overall growth performance. A strong positive genetic correlation was observed between total purine and guanine content (0.87 and 0.76 in liver and breast meat) for various biochemical traits, indicating that these traits are genetically closely linked. The uric acid levels in breast meat and liver showed a significant positive genetic correlation with guanine content (0.40 and 0.48, respectively) and SOD activity (0.64 and 0.78, respectively). This finding suggests that higher guanine content and SOD activity were associated with increased uric acid levels in both breast meat and liver. Additionally, the genetic correlations between uric acid levels and other purine contents were positive, except for adenine, which demonstrated a negative relationship. Negative genetic correlations were found between purine content traits (total, adenine, guanine, xanthine, and hypoxanthine) and BW traits at various stages, with correlation coefficients ranging from −0.11 to −0.82. This suggested a genetic tradeoff between purine content and growth traits. The correlation between xanthine and hypoxanthine contents was low (0.01), indicating that these traits were genetically independent. This study also found that SOD activity was positively correlated with uric acid levels (0.71), indicating that higher SOD activity is genetically associated with increased uric acid levels. Phenotypic correlations between total purine content and BW in native Thai chickens were predominantly negative. A significant positive correlation was observed between the guanine content and BW at different growth stages. Adenine content was negatively correlated with ADG from 0–2 and 2–4 weeks. Uric acid levels in breast meat positively correlated with BW at 6, 8, and 10 weeks. SOD activity in the blood was positively correlated with BW and average daily weight gain during later growth periods. This study revealed that xanthine content had a positive phenotypic correlation with BW at 4, 6, and 8 weeks. Hypoxanthine content was negatively correlated with BW and ADG across all measured growth periods.

## 4. Discussion

Over several decades, the primary goal of genetic selection has been to enhance production traits, such as growth rate, feed efficiency, and meat yield for consumption. Potue et al. [6] reported efforts to breed native chickens to improve meat quality and reduce purine content in the KKU-ONE chicken breed, a crossbred Thai native. In this study, we evaluated the growth efficiency of Thai native chickens (Shee) in comparison to another Thai native chicken breed, Pradu Hang Dum. According to Boonkum et al. [18], Pradu Hang Dum chickens achieved a final body weight (BW) of approximately 1500 g at 10 weeks of age, which is lower than the final BW observed for the Shee chickens in the present study. Regarding purine content, the levels at 6, 8, and 10 weeks of age decreased with age, which was consistent with the results from a study on Korat chickens by Kubota et al. [22]. They found the highest purine content at 2 weeks, indicating considerable variation in purine content at different ages. The average purine content in breast meat was 158.1 mg/100 g, and the calculated uric acid content was 191.0 mg/100 g. This is similar to the findings of Kaneko et al. [21], who reported that chicken breast meat has moderate purine content, ranging from 100 to 200 mg/100 g. Potue et al. [6] also reported differences in purine content among chicken breeds, with the lowest amount found in other breed Thai native chickens (Pradu Hang Dum) at 126.83 mg/100 g compared to that in broiler chickens (178.92 mg/100 g) and crossbred Thai native chickens (155.79 mg/100 g). We found purine derivatives (adenine, guanine, and hypoxanthine) values in Thai native chickens (Shee) similar to those reported by Potue et al. [6] in Thai native chickens (Pradu Hang Dum). In our study, xanthine content in breast meat was low (2.09–2.44 mg/100 g), whereas Potue et al. [6] did not detect xanthine in Thai native chickens (Pradu Hang Dum). Chicken meat contains 141.2 mg/100 g and 171.8 mg/100 g. These values are approximately 1.55 times higher than those observed in pork (284.8 mg/100 g) and beef (219.8 mg/100 g) [21]. Chickens accumulate more purines and uric acid than mammals because they lack uricase, which breaks down uric acid into allantoin. In contrast, mammals possess the enzyme uricase [25]. In this study, the liver of chickens aged 6–10 weeks had an average purine content of 323.2 mg/100 g and a calculated uric acid content of 377.8 mg/100 g, similar to the report by Kaneko et al. [21], which found a purine content of 312.2 mg/100 g and a calculated uric acid content of 365.1 mg/100 g in broilers.

In addition to purines, its derivatives contain adenine, guanine, hypoxanthine, and xanthine. We found hypoxanthine in breast meat at a proportion as high as 54% of the total purine content, which was consistent with Kaneko et al. [21], who reported that more than 50% of the total purine content in meat is hypoxanthine. The relatively high proportion of hypoxanthine had a greater effect on uric acid content than the other derivatives [20]. The liver contained 35% adenine and 42% guanine relative to the total purine content in native chickens aged 6–10 weeks. Previous studies have suggested that high levels of uric acid in the blood are not caused by the consumption of foods high in purines but rather by certain purine derivatives, including hypoxanthine and adenine, depending on the source of chicken parts [21,26]. This implied that the focus should not only be on purine content but also on purine derivatives. However, our study focused exclusively on slow-growing Thai native chickens to provide insights specific to their genetic improvement. The inclusion of fast-growing genotypes as a control group was beyond the scope of this study. However, previous research has extensively demonstrated that fast-growing chickens exhibit higher purine levels, primarily due to their elevated protein turnover and metabolic activity [1,4,5]. These findings align with our hypothesis that fast-growing genotypes are likely to have higher purine content. Future studies could expand on this work by incorporating fast-growing chickens as a comparative group to further validate these relationships and provide a more comprehensive understanding in a broader genetic and metabolic context.

We found that the blood SOD levels at 6 weeks of age were higher than those at 8 and 10 weeks, with values of 8.16, 6.99, and 5.88 U/mL, respectively. These values were within the range reported for purebred native chickens aged 6–10 weeks, which was between 4.88 and 7.08 during the same period [11]. SOD levels appeared to decrease with increasing age, which was consistent with the findings of Tantiyasawasdikul et al. [11] who reported that blood SOD levels decreased with age. In addition to age, blood SOD levels in crossbred chickens are influenced by various factors, with purebred Thai natives having higher SOD levels than crossbred Thai native chickens. Chicken breeds selected for their high growth rates and breast meat production are more sensitive to oxidative stress. However, birds have an antioxidant defense system that develops during the embryonic stage, helping them maintain a redox balance at the cellular and tissue levels [27,28,29]. During growth, SOD activity remained high in both plasma and meat, with slow-growing chickens displaying greater stability than fast-growing broilers. SOD plays an important role in maintaining the redox balance in slow-growing chickens, while fast-growing broilers maintain high growth rates due to high protein turnover, as indicated by the plasma uric acid content from hatching until 3 weeks of age [27].

The heritability estimates for growth traits BW, ADG, and BrC in Thai native chickens were moderate to high (0.27–0.48; see Table 3). This range was similar to the heritability estimates for growth traits in Thai native chickens reported by Chaikuad et al. [30], which ranged from 0.20 to 0.49. The heritability estimates for BW in our study were moderate, consistent with that of several previous studies on local Venda chickens [31], Mazandaran native chickens [32], and various breeds of Thai native chickens [30,33,34]. For the biweekly ADG, our study reported small variations within a moderate range. This contrasted with previous reports on Korean native chickens, where heritability estimates ranged widely from low to medium (0.08–0.48), according to Manjula et al. [35]. The moderate-to-high heritability of growth traits implies that a genetic response can be achieved through selection. The related traits tended to decrease with age, which was consistent with the findings of Manjula et al. [35]. Improving growth traits to enhance the genetic performance of native chickens can be achieved by selecting early growth traits. However, the first hatch weight had relatively low direct heritability estimates, possibly because of the interference of maternal genetic effects, which had a relatively high proportion [33].

Genetic parameters of purine content have rarely been reported. Therefore, our study is one of the first to report heritability estimates for purine content in chickens. Our results showed that purine content had low heritability in the breast meat and liver (0.05–0.21). The guanine and total purine contents in the liver had the highest heritability compared to other purine contents and those assessed from breast meat across all ages of chickens (0.134–0.190; see Table 4). Heritability estimates of SOD activity in the blood at 6, 8, and 10 weeks of age were moderate, varying from 0.27 to 0.29. According to Zhang et al. [36], the heritability of blood chemical parameters in broiler chickens varies from 0.26 to 0.60. The presence of genetic variations in purine characteristics and SOD activity in native chickens indicated the possibility of selectively breeding Thai native chickens into low-purine breeds. The difference in purine composition between various chicken tissues (liver and breast) was consistent with previous reports by Tantiyasawasdikul et al. [11]. Breast meat data at 10 weeks of age suggest that this is a suitable point for genetic evaluation, as it corresponds to the market weight of native chickens.

In this study, genetic and phenotypic correlation analysis revealed a negative relationship between purine content (total purine, adenine, and guanine) and growth performance. This relationship strengthens with increasing BW, as reported by Tantiyasawasdikul et al. [11]. Conversely, hypoxanthine, which accounts for more than 50% of the total purine content in meat, was weakly correlated with growth performance. Regarding the relationship between growth and purine content and its derivatives, previous studies reported that among different breeds of Thai chickens of the same age, Mae Hong Son chickens had lower BW and lower purine content than Thai native chickens (Pradu Hang Dum). Moreover, when Thai native chickens were compared with fast-growing broiler chickens, the levels of adenine, guanine, hypoxanthine, total purine, and uric acid in the breast meat were significantly lower [7,13]. This relationship between growth and purine content may be related to purine nucleotide biosynthesis, which involves ATP production and promotes an increase in the growth rate [37]. In genetic breeding plan, the importance of multi-trait selection indices to balance traits. For example, if growth and a negatively correlated biochemical trait are both desirable, weights can be assigned to each trait to optimize overall genetic improvement.

Furthermore, we found positive genetic correlations for all growth traits including BW, biweekly ADG, and BrC (Table 5). A strong positive genetic correlation was observed between BW and BrC at 6–10 weeks of age (0.68 0.97). These results indicated a strong genetic relationship between growth performance and meat quality traits in chickens.

The genetic correlation in this study revealed an interesting relationship between purine content and growth performance, showing a negative genetic correlation that has not been observed in previous studies reporting genetic relationships for these traits. However, de Verdal et al. [38] reported that nitrogen, a component of purine, was negatively correlated with breast yield and nitrogen retention in chickens (−0.33). This suggested that focusing on genetic improvements in growth and meat production may lead to increased purine content, highlighting the need to consider these characteristics together if the goal is to genetically improve fast-growing, low-purine chickens. Protein accumulation was linked to nitrogen retention efficiency but not to intake or excretion. When studying purine content, it was discovered that the genetic correlation between guanine and total purines was strongly positive. Zheng et al. [39] observed that the concentration of guanine molecules was the primary cause of the differences in total purine content. Therefore, the genetic correlation between guanine and total purines may be higher than that between other purine components. Excessive uric acid production is caused by purine metabolism [40]. A previous study reported that uric acid has a high genetic correlation (−0.57 to −0.60) with abdominal fat characteristics in broiler chickens [36].

The positive correlation between SOD activity and the total purine, adenine, guanine, hypoxanthine, and uric acid levels in chicken meat indicates that higher purine levels contribute to the production of more antioxidant defense systems [11,27]. It was commonly recognized that SOD and uric acid had a relationship in metabolism. Batool et al. [41] reported that in hyperuricemic rats, SOD concentration also increased with increasing uric acid concentration. We found a moderate positive genetic association between SOD and total purines and uric acid (0.49 and 0.67, respectively). Physiologically, it was commonly recognized that SOD and purines to uric acid had a metabolic relationship. This relationship occurs because of the degradation of purine nucleotides to xanthine and hypoxanthine and later to uric acid via xanthine oxidase (XO). During this process, free radicals, especially superoxide anions (O^2−^), are created and require SOD activity for conversion to hydrogen peroxide (H_2_O_2_) [40,41,42,43].

The high positive genetic correlation (0.80 to 0.95) between BW at 6–10 weeks and BrC at the same age indicated that favorable growth traits could promote the genetic development of common meat quality traits. Selecting BW at 6 weeks of age is an early and accurate method for sex sorting and is suitable for breeding management to improve both growth and meat content. Furthermore, the negative correlation between chickens with higher BW, meat content, and purine content indicates that this selection did not result in increased purine levels. Similarly, there was a positive correlation between BW at 6 weeks and SOD activity and uric acid levels, which act as antioxidants but must be balanced by regulating purine production. Using a selection index for growth and meat quality based on BW at 6 weeks while selecting for lower SOD and uric acid levels was a strategy to help develop chickens for functional food. The aim of this approach was to improve the genetics of chickens with good growth and low purine content.

## 5. Conclusions

This study highlights the strengths of using a multi-trait animal model, which demonstrated high efficiency in estimating genetic parameters. The research focused on investigating the genetic parameters and correlations among growth traits, purine content, uric acid levels, and superoxide dismutase (SOD) activity in Thai native chickens. The results revealed moderate heritability for growth traits and low to moderate heritability for biochemical traits, indicating the feasibility of targeted genetic selection. Additionally, the study identified negative genetic correlations between purine content and growth traits, providing valuable insights for developing balanced breeding programs. Nevertheless, challenges persist in simultaneously enhancing the genetic potential of Thai native chickens for both growth and biochemical traits.

## Figures and Tables

**Table 1:** Descriptive statistics of growth traits in Thai native chickens (mean ± SD).

Traits	Number of Samples	Growth Traits
BW0	300	35.47 ± 3.31 ^f^
BW2	300	186.67 ± 13.43 ^e^
BW4	300	530.06 ± 34.17 ^d^
BW6	300	951.17 ± 50.66 ^c^
BW8	270	1387.69 ± 119.29 ^b^
BW10	240	1834.56 ± 198.67 ^a^
ADG0–2	300	23.63 ± 2.64 ^ab^
ADG2–4	300	21.80 ± 1.44 ^b^
ADG4–6	300	24.43 ± 2.15 ^a^
ADG6–8	270	27.97 ± 2.63 ^a^
ADG8–10	240	25.10 ± 2.81 ^a^
BrC6	300	23.01 ± 0.63 ^c^
BrC8	270	25.26 ± 0.72 ^b^
BrC10	240	28.87 ± 0.83 ^a^

BW0 = hatching weight; BW2, BW4, BW6, BW8, and BW10 = body weight at 2, 4, 6, 8, and 10 weeks of age; ADG0–2, ADG2–4, ADG4–6, ADG6–8, and ADG8–10 = average daily gain at 0–2, 2–4, 4–6, 6–8, and 8–10 weeks of age; BrC6, BrC8, and BrC10 = breast circumference at 6, 8, and 10 weeks. ^a, b, c, d, e, f^ = different superscript letters indicate significant differences within the column for each parameter at *p* < 0.05.

**Table 2:** Descriptive statistics of purine content, uric acid in breast meat and liver, and SOD in the blood of Thai native chickens (mean ± SD).

Traits	Number of Samples	Growth Traits	Purine Contents in Breast Meat	Purine Contents in Liver
Total purine (mg/100 g)				
6 wk	30	-	163.48 ± 6.48 ^a,y^	347.77 ± 12.92 ^a,x^
8 wk	30	-	159.92 ± 5.22 ^ab,y^	324.21 ± 12.38 ^ab,x^
10 wk	30	-	151.00 ± 5.47 ^b,y^	297.62 ± 12.05 ^b,x^
Adenine (mg/100 g)				
6 wk	30	-	35.18 ± 1.52 ^a,y^	124.11 ± 6.45 ^a,x^
8 wk	30	-	33.84 ± 1.62 ^ab,y^	116.08 ± 5.71 ^ab,x^
10 wk	30	-	30.70 ± 1.63 ^b,y^	105.79 ± 5.82 ^b,x^
Guanine (mg/100 g)				
6 wk	30	-	37.28 ± 1.70 ^a,y^	146.20 ± 8.95 ^a,x^
8 wk	30	-	36.62 ± 1.78 ^a,y^	137.62 ± 8.37 ^ab,x^
10 wk	30	-	35.42 ± 1.67 ^a,y^	131.79 ± 7.82 ^b,x^
Xanthine (mg/100 g)				
6 wk	30	-	2.44 ± 0.53 ^a,y^	60.95 ± 3.74 ^a,x^
8 wk	30	-	2.26 ± 0.52 ^ab,y^	55.57 ± 3.87 ^ab,x^
10 wk	30	-	2.09 ± 0.40 ^a,y^	47.62 ± 3.48 ^b,x^
Hypoxanthine (mg/100 g)				
6 wk	30	-	88.58 ± 4.32 ^a,x^	16.52 ± 2.55 ^a,y^
8 wk	30	-	87.21 ± 4.42 ^a,x^	14.94 ± 2.22 ^ab,y^
10 wk	30	-	82.79 ± 3.15 ^b,x^	12.43 ± 2.39 ^b,y^
Calculated as uric acid (mg/100 g)				
6 wk	30	-	197.33 ± 4.82 ^a,y^	405.86 ± 6.13 ^a,x^
8 wk	30	-	192.61 ± 4.57 ^ab,y^	379.92 ± 5.41 ^b,x^
10 wk	30	-	183.18 ± 4.39 ^b,y^	347.63 ± 5.43 ^c,x^
SOD in blood (U/mL)				
6 wk	30	8.16 ± 0.49 ^a^	-	-
8 wk	30	6.99 ± 0.41 ^b^	-	-
10 wk	30	5.88 ± 0.36 ^c^	-	-

Calculated as uric acid (mg/100 g) = $\frac{\mathrm{Total}\;\mathrm{purine}\;\left(\mu \mathrm{mol}/100\;g\right)\times 168.1}{1000}$ when the molecular weights of adenine, guanine, xanthine, hypoxanthine, and uric acid = 135.1, 151.1, 136.1, 152.1, and 168.1, respectively. ^a, b, c^ = different superscript letters indicate significant differences within the column for each parameter at *p* < 0.05. ^x, y^ = different superscript letters differ significantly within rows for each parameter at *p* < 0.05.

**Table 3:** Variance components and estimated heritability of growth traits of Thai native chickens.

Traits	Additive Variance	Residual Variance	Heritability (±SE)
Body weight (BW; g)			
BW0	4.99	5.30	0.485 ± 0.001
BW2	251.05	400.98	0.385 ± 0.002
BW4	522.25	1029.28	0.337 ± 0.003
BW6	3500.50	6976.00	0.334 ± 0.003
BW8	3625.07	7889.28	0.315 ± 0.004
BW10	11,541.00	26,410.00	0.304 ± 0.005
Average daily gain (ADG; g/day)			
ADG0–2	1.15	2.28	0.335 ± 0.001
ADG2–4	1.05	2.56	0.291 ± 0.002
ADG4–6	1.64	4.10	0.286 ± 0.003
ADG6–8	1.95	5.20	0.273 ± 0.004
ADG8–10	2.35	6.44	0.270 ± 0.004
Breast circumference (BrC; cm)			
BrC6	0.48	1.05	0.314 ± 0.003
BrC8	0.53	1.33	0.285 ± 0.005
BrC10	0.66	1.65	0.286 ± 0.005

BW0 = hatching weight; BW2, BW4, BW6, BW8, and BW10 = body weight at 2, 4, 6, 8, and 10 weeks of age; ADG0–2, ADG2–4, ADG4–6, ADG6–8, and ADG8–10 = average daily gain at 0–2, 2–4, 4–6, 6–8, and 8–10 weeks of age; BrC6, BrC8, and BrC10 = breast circumference at 6, 8, and 10 weeks.

**Table 4:** Variance components and estimated heritability of purine content, uric acid in breast meat and liver, and SOD in the blood of Thai native chickens.

Traits	Breast Meat			Liver		
	Additive Variance	Residual Variance	Heritability (±SE)	Additive Variance	Residual Variance	Heritability (±SE)
Total purine (mg/100 g)						
6 week	1421.00	12,220.25	0.104 ± 0.005	2487.00	10,630.88	0.190 ± 0.006
8 week	1405.12	12,235.46	0.103 ± 0.005	2477.65	10,635.42	0.189 ± 0.006
10 week	1400.77	12,210.11	0.103 ± 0.006	2472.12	10,639.46	0.189 ± 0.007
Adenine (mg/100 g)						
6 week	73.78	786.00	0.086 ± 0.002	256.34	1965.00	0.115 ± 0.005
8 week	71.01	790.12	0.082 ± 0.002	248.03	1975.30	0.112 ± 0.004
10 week	69.44	792.86	0.081 ± 0.002	243.32	1982.15	0.109 ± 0.004
Guanine (mg/100 g)						
6 week	119.00	714.00	0.143 ± 0.003	626.80	2846.00	0.180 ± 0.005
8 week	115.44	720.35	0.138 ± 0.003	600.19	2868.56	0.173 ± 0.005
10 week	112.56	725.13	0.134 ± 0.003	582.49	2891.67	0.168 ± 0.005
Xanthine (mg/100 g)						
6 week	2.43	64.86	0.036 ± 0.002	15.22	200.14	0.071 ± 0.003
8 week	2.40	64.99	0.036 ± 0.002	14.15	212.79	0.062 ± 0.003
10 week	2.38	65.01	0.035 ± 0.002	13.08	220.24	0.056 ± 0.003
Hypoxanthine (mg/100 g)						
6 week	25.22	222.30	0.102 ± 0.003	5.19	75.24	0.069 ± 0.003
8 week	25.15	225.11	0.100 ± 0.003	5.02	88.44	0.057 ± 0.003
10 week	25.08	229.89	0.098 ± 0.003	4.81	95.74	0.050 ± 0.003
Calculated as uric acid (mg/100 g)						
6 week	2715.01	19,973.12	0.120 ± 0.004	6842.00	25,245.00	0.213 ± 0.007
8 week	2710.25	19,999.00	0.119 ± 0.004	6374.85	25,477.85	0.200 ± 0.008
10 week	2704.89	20,011.84	0.119 ± 0.004	5782.71	25,652.12	0.184 ± 0.007
SOD in blood (U/mL)						
6 week	0.20	0.49	0.290 ± 0.003	-	-	-
8 week	0.20	0.52	0.278 ± 0.003	-	-	-
10 week	0.19	0.52	0.268 ± 0.003	-	-	-

**Table 5:** Genetic correlation between growth traits, purine content, uric acid in breast meat (above diagonal) and liver (below diagonal), and SOD in the blood of Thai native chickens.

Traits	Total	Aden	Guan	Xant	Hypo	Uric	SOD	BW0	BW2	BW4	BW6	BW8	BW10	ADG 0–2	ADG 2–4	ADG 4–6	ADG 6–8	ADG 8–10	BrC 6	BrC 8	BrC 10
Total	-	0.29	0.76	0.44	0.26	0.31	0.49	−0.11	−0.29	−0.38	−0.40	−0.41	−0.48	−0.17	−0.39	−0.39	−0.45	−0.51	−0.42	−0.42	−0.54
Aden	0.42	-	0.11	0.20	0.46	−0.27	−0.25	−0.13	−0.29	−0.37	−0.42	−0.45	−0.49	−0.25	−0.32	−0.38	−0.37	−0.44	−0.29	−0.35	−0.42
Guan	0.87	0.24	-	0.23	0.16	0.40	0.45	−0.13	−0.19	−0.24	−0.28	−0.33	−0.36	−0.16	−0.22	−0.34	−0.41	−0.43	−0.24	−0.34	−0.38
Xant	0.56	0.39	0.38	-	0.01	0.19	−0.30	0.05	0.08	0.10	0.17	0.20	0.22	0.07	0.09	0.17	0.25	0.28	0.19	0.28	0.36
Hypo	0.30	0.20	0.29	0.14	-	0.20	0.24	−0.11	−0.24	−0.36	−0.43	−0.58	−0.66	−0.19	−0.38	−0.44	−0.55	−0.77	−0.35	−0.36	−0.38
Uric	0.41	−0.46	0.48	0.24	0.27	-	0.64	0.14	0.22	0.40	0.65	0.78	0.82	0.21	0.35	0.49	0.70	0.78	0.40	0.46	0.69
SOD	0.64	−0.35	0.67	0.08	0.32	0.78	-	0.14	0.30	0.42	0.53	0.64	0.71	0.25	0.48	0.56	0.72	0.88	0.30	0.38	0.45
BW0	−0.29	−0.18	−0.15	0.18	0.04	0.25	0.14	-	0.25	0.18	0.14	0.09	0.05	0.32	0.28	0.22	0.15	0.08	0.22	0.15	0.08
BW2	−0.42	−0.46	−0.25	0.27	0.06	0.44	0.30	0.25	-	0.73	0.68	0.60	0.54	0.68	0.61	0.55	0.52	0.46	0.35	0.30	0.26
BW4	−0.45	−0.51	−0.32	0.25	0.07	0.59	0.42	0.18	0.73	-	0.82	0.79	0.71	0.79	0.85	0.80	0.75	0.71	0.52	0.62	0.48
BW6	−0.49	−0.62	−0.35	0.26	0.06	0.78	0.53	0.14	0.68	0.82	-	0.88	0.82	0.75	0.82	0.88	0.85	0.80	0.95	0.90	0.86
BW8	−0.52	−0.69	−0.36	0.39	0.06	0.82	0.64	0.09	0.60	0.79	0.88	-	0.94	0.72	0.79	0.82	0.91	0.85	0.92	0.96	0.93
BW10	−0.64	−0.75	−0.38	0.39	0.05	0.86	0.71	0.05	0.54	0.71	0.82	0.94	-	0.70	0.74	0.79	0.88	0.95	0.90	0.95	0.98
ADG 0–2	−0.37	−0.48	−0.22	0.26	0.07	0.30	0.25	0.32	0.68	0.79	0.75	0.72	0.70	-	0.85	0.82	0.80	0.79	0.75	0.72	0.71
ADG 2–4	−0.49	−0.52	−0.30	0.37	0.09	0.52	0.48	0.28	0.61	0.85	0.82	0.79	0.74	0.85	-	0.85	0.84	0.82	0.80	0.78	0.77
ADG 4–6	−0.58	−0.55	−0.35	0.34	0.08	0.64	0.56	0.22	0.55	0.80	0.88	0.82	0.79	0.82	0.85		0.88	0.87	0.86	0.88	0.87
ADG 6–8	−0.62	−0.60	−0.42	0.38	0.08	0.85	0.72	0.15	0.52	0.75	0.85	0.91	0.88	0.80	0.84	0.88	-	0.91	0.94	0.96	0.96
ADG 8–10	−0.74	−0.82	−0.48	0.41	0.09	0.90	0.88	0.08	0.46	0.71	0.80	0.85	0.95	0.79	0.82	0.87	0.91	-	0.97	0.98	0.99
BrC6	−0.49	−0.45	−0.35	0.38	0.06	0.45	0.30	0.22	0.35	0.52	0.95	0.92	0.90	0.75	0.80	0.86	0.94	0.97	-	0.96	0.95
BrC8	−0.56	−0.56	−0.38	0.46	0.08	0.51	0.38	0.15	0.30	0.62	0.90	0.96	0.95	0.72	0.78	0.88	0.96	0.98	0.96	-	0.97
BrC10	−0.67	−0.69	−0.41	0.49	0.10	0.78	0.45	0.08	0.26	0.48	0.86	0.93	0.98	0.71	0.77	0.87	0.96	0.99	0.95	0.97	-

Total = Total purine; Aden = Adenine; Guan = Guanine; Xant = Xanthine; Hypo = Hypoxanthine; Uric = uric acid; SOD = superoxide dismutase; BW0 = hatching weight; BW2, BW4, BW6, BW8, and BW10 = body weight at 2, 4, 6, 8, and 10 weeks of age; ADG0–2, ADG2–4, ADG4–6, ADG6–8, and ADG8–10 = average daily gain during 0–2, 2–4, 4–6, 6–8, and 8–10 weeks of age; BrC6, BrC8, and BrC10 = breast circumference at 6, 8, and 10 weeks of age.

**Table 6:** Phenotypic correlation between growth traits, purine content, uric acid in breast meat (above diagonal) and liver (below diagonal), and SOD in the blood of Thai native chickens.

Traits	Total	Aden	Guan	Xant	Hypo	Uric	SOD	BW0	BW2	BW4	BW6	BW8	BW10	ADG 0–2	ADG 2–4	ADG 4–6	ADG 6–8	ADG 8–10	BrC 6	BrC 8	BrC 10
Total	-	0.40	0.86	0.56	0.37	0.44	0.59	−0.22	−0.35	−0.49	−0.52	−0.53	−0.60	−0.29	−0.50	−0.51	−0.55	−0.61	−0.55	−0.52	−0.62
Aden	0.55	-	0.25	0.30	0.55	−0.38	−0.36	−0.23	−0.40	−0.45	−0.56	−0.59	−0.61	−0.35	−0.42	−0.50	−0.50	−0.55	−0.40	−0.46	−0.55
Guan	0.94	0.38	-	0.32	0.29	0.50	0.55	−0.33	−0.39	−0.39	−0.38	−0.40	−0.46	−0.28	−0.33	−0.45	−0.52	−0.55	−0.46	−0.56	−0.50
Xant	0.66	0.50	0.44	-	0.12	0.28	−0.40	0.15	0.19	0.22	0.30	0.30	0.35	0.17	0.20	0.29	0.37	0.40	0.31	0.40	0.48
Hypo	0.42	0.33	0.38	0.25	-	0.30	0.34	−0.22	−0.36	−0.48	−0.55	−0.60	−0.72	−0.31	−0.50	−0.56	−0.67	−0.89	−0.47	−0.48	−0.50
Uric	0.61	−0.59	0.59	0.34	0.38	-	0.72	0.25	0.33	0.50	0.75	0.88	0.88	0.33	0.47	0.61	0.80	0.88	0.52	0.58	0.71
SOD	0.72	−0.47	0.72	0.19	0.44	0.88	-	0.24	0.40	0.52	0.55	0.74	0.82	0.36	0.59	0.62	0.84	0.92	0.45	0.50	0.57
BW0	−0.39	−0.32	−0.30	0.28	0.19	0.39	0.29	-	0.25	0.25	0.20	0.17	0.15	0.42	0.38	0.32	0.25	0.18	0.32	0.35	0.18
BW2	−0.55	−0.55	−0.39	0.39	0.20	0.59	0.40	0.31	-	0.80	0.78	0.70	0.64	0.78	0.71	0.65	0.62	0.55	0.42	0.42	0.39
BW4	−0.67	−0.67	−0.48	0.40	0.18	0.65	0.51	0.28	0.83	-	0.85	0.89	0.82	0.88	0.91	0.90	0.85	0.81	0.62	0.72	0.68
BW6	−0.61	−0.78	−0.49	0.42	0.19	0.88	0.62	0.26	0.75	0.88	-	0.98	0.92	0.88	0.92	0.92	0.97	0.90	0.97	0.94	0.96
BW8	−0.66	−0.78	−0.49	0.51	0.18	0.89	0.75	0.22	0.77	0.85	0.92	-	0.98	0.85	0.89	0.92	0.95	0.95	0.94	0.97	0.96
BW10	−0.78	−0.81	−0.48	0.53	0.15	0.95	0.81	0.18	0.66	0.79	0.87	0.98	-	0.80	0.84	0.89	0.95	0.98	0.92	0.96	0.99
ADG 0–2	−0.52	−0.59	−0.29	0.44	0.19	0.42	0.38	0.45	0.79	0.81	0.80	0.82	0.80	-	0.94	0.92	0.90	0.89	0.85	0.82	0.81
ADG 2–4	−0.67	−0.65	−0.42	0.49	0.20	0.65	0.59	0.39	0.72	0.90	0.85	0.85	0.85	0.95	-	0.94	0.85	0.93	0.91	0.89	0.90
ADG 4–6	−0.71	−0.69	−0.48	0.49	0.21	0.74	0.67	0.33	0.68	0.90	0.92	0.88	0.88	0.88	0.95		0.95	0.97	0.96	0.98	0.97
ADG 6–8	−0.78	−0.73	−0.55	0.51	0.21	0.91	0.80	0.28	0.69	0.85	0.88	0.92	0.92	0.85	0.94	0.98	-	0.95	0.96	0.97	0.98
ADG 8–10	−0.85	−0.88	−0.59	0.55	0.22	0.98	0.95	0.20	0.59	0.79	0.85	0.90	0.98	0.89	0.92	0.97	0.98	-	0.99	0.99	0.99
BrC6	−0.60	−0.59	−0.49	0.50	0.19	0.59	0.44	0.35	0.49	0.68	0.98	0.95	0.92	0.85	0.90	0.96	0.95	0.99	-	0.99	0.99
BrC8	−0.69	−0.67	−0.48	0.60	0.22	0.67	0.58	0.28	0.42	0.66	0.92	0.98	0.98	0.82	0.88	0.98	0.98	0.99	0.98	-	0.99
BrC10	−0.79	−0.79	−0.55	0.61	0.25	0.89	0.59	0.20	0.40	0.55	0.89	0.95	0.99	0.81	0.87	0.97	0.99	0.99	0.99	0.99	-

Total = Total purine; Aden = Adenine; Guan = Guanine; Xant = Xanthine; Hypo = Hypoxanthine; Uric = uric acid; SOD = superoxide dismutase; BW0 = hatching weight; BW2, BW4, BW6, BW8, and BW10 = body weight at 2, 4, 6, 8, and 10 weeks of age; ADG0–2, ADG2–4, ADG4–6, ADG6–8, and ADG8–10 = average daily gain during 0–2, 2–4, 4–6, 6–8, and 8–10 weeks of age; BrC6, BrC8, and BrC10 = breast circumference at 6, 8, and 10 weeks of age.

## Data Availability

The original contributions presented in the study are included in the article, further inquiries can be directed to the corresponding author.
